# Combinatorial Codes and Labeled Lines: How Insects Use Olfactory Cues to Find and Judge Food, Mates, and Oviposition Sites in Complex Environments

**DOI:** 10.3389/fphys.2018.00049

**Published:** 2018-02-01

**Authors:** Alexander Haverkamp, Bill S. Hansson, Markus Knaden

**Affiliations:** Department of Evolutionary Neuroethology, Max Planck Institute for Chemical Ecology, Jena, Germany

**Keywords:** olfaction, *Drosophila*, mosquito, moth, labeled line, odor background

## Abstract

Insects, including those which provide vital ecosystems services as well as those which are devastating pests or disease vectors, locate their resources mainly based on olfaction. Understanding insect olfaction not only from a neurobiological but also from an ecological perspective is therefore crucial to balance insect control and conservation. However, among all sensory stimuli olfaction is particularly hard to grasp. Our chemical environment is made up of thousands of different compounds, which might again be detected by our nose in multiple ways. Due to this complexity, researchers have only recently begun to explore the chemosensory ecology of model organisms such as *Drosophila*, linking the tools of chemical ecology to those of neurogenetics. This cross-disciplinary approach has enabled several studies that range from single odors and their ecological relevance, via olfactory receptor genes and neuronal processing, up to the insects' behavior. We learned that the insect olfactory system employs strategies of combinatorial coding to process general odors as well as labeled lines for specific compounds that call for an immediate response. These studies opened new doors to the olfactory world in which insects feed, oviposit, and mate.

## Introduction

While flying or walking through its natural environment any insect, be it a mosquito, a fly or a giant sphinx moth, encounters a nearly infinite number of chemical signals. These chemical messages differ widely in the specificity at which they target potential receivers as well as in their signal complexity (Junker et al., 2017). Some chemical signals, like insect sex pheromones for example target only conspecifics of the opposite sex, while a flower might aim to attract as many pollinators as possible with its odor bouquet. In parallel, a single compound might be enough for an insect to identify a toxic food source while in other cases an entire blend of compounds might be required to identify a suitable oviposition site. These different types of chemical messages, however, do not only arise in the way the message is produced by the sender, but also by the way these messages are coded on the sensory periphery and in the brain of the receiver. Over the last years, great progress has been made to decipher the code by which insects read the chemical messages of their environment using neurobiological and ethological methods (Hansson and Stensmyr, 2011). These methods form the cornerstone of this review in which we approach the neuronal computation, namely labeled lines, and combinatorial coding, used by insects while they are foraging and/or searching for oviposition sites or mates. We discuss the different systems by which insects might detect specific odors of significant importance or multiple odors in a combinatorial system within their environment.

## The neuronal basis of insect olfaction

**Figure 1:**
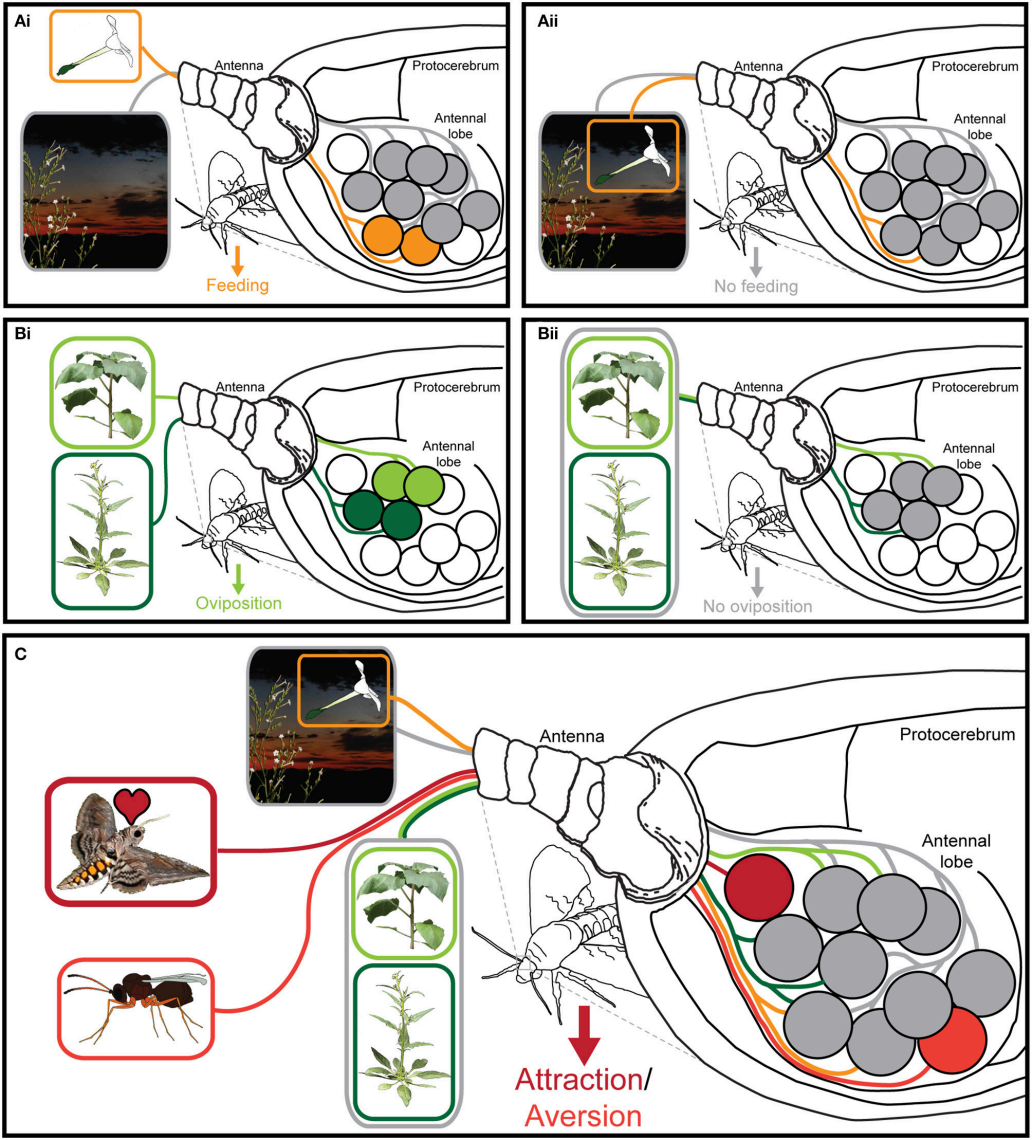
Processing of olfactory information in the insect antennal lobe. **(Ai)** Insects can easily navigate toward an odor source (orange), in front of a chemically distinct odor background. **(Aii)** This navigation however becomes corrupted, when the same volatiles are present both in the background as well as in the odor source (gray) (Riffell et al., 2014). **(Bi)** When the odor blends of two different host plants were presented spatially separated, both were attractive to the female moth (green), but **(Bii)** when these two blends were mixed both spatially and temporally into a novel mixture, this new blend became meaningless or even repellent to the moth (gray) (Spaethe et al., 2013). **(C)** Ecologically labeled lines (red) are processed independently of background odors or other odor plumes present in the environment of the insect (Badeke et al., 2016).

Due to the existence of comprehensive reviews on the insect olfactory system (de Bruyne and Baker, 2008; Hansson and Stensmyr, 2011), let us just briefly summarize: of all sensory stimuli, scent, and taste are the hardest to capture. Contrary to the visual and auditory systems that basically deal with wavelengths and amplitudes of stimuli along a linear scale, chemical senses detect stimuli, which might vary on dozens of properties, such as chain length, polarity, chirality, and many more. This multitude of properties presumably explains why the olfactory system requires many more different receptor types than for example the visual system. Insects detect volatile molecules with olfactory sensory neurons (OSNs) expressing either olfactory receptors (Ors), ionotropic receptors (Irs), or (rarely) gustatory receptors (Grs) (for a review on the function of chemosensory receptors see Touhara and Vosshall, 2009). OSNs expressing the same receptor project to the same spherical structure (called glomerulus) within the first olfactory processing center, the antennal lobe. Within the antennal lobe, OSNs connect to local interneurons and projection neurons that convey the olfactory information to higher brain centers like the mushroom bodies and the lateral horn. In the winged insects the number of described olfactory receptors ranges from 10 in lice (Kirkness et al., 2011) to ca 375 in some leafcutter ants (Zhou et al., 2015). The identity of the expressed Or (individual OSNs usually express only one Or gene) dictates to which volatiles an OSN will respond. From studies in the model *Drosophila melanogaster*, where most Ors have been deorphanized (Hallem and Carlson, 2006), we know that many receptors are broadly tuned and contribute to the so-called olfactory code: an OSN expressing a broadly tuned receptor detects several dozens of different compounds, and a specific compound can activate OSNs expressing different receptors. Hence, odor identity is coded by the activation of a subpopulation of those OSNs whose receptors interact with this odor. To complicate matters further, a certain odor source such as a flower or a ripening fruit, might emit a great variety of odors, detected by these broadly tuned receptors, resulting in the so-called “olfactory Gestalt” of the odor source (Dethier, 1982; Figures 1A,B). However, more recent investigations have shown that odors of outstanding ecological relevance are often detected by OSNs expressing highly selective Ors. *Drosophila* for example expresses Ors that are narrowly tuned to sex pheromones, or food and oviposition cues. For example, DmOr67d (each *Drosophila* Or is numbered based on the position of its gene within the *Drosophila* genome) detects 11-cis-vaccenyl acetate, a male produced pheromone that becomes transferred to females during copulation and inhibits further mating of the female (Kurtovic et al., 2007). DmOr47b is tuned to methyl laurate (and—following Lin et al.—also to palmitoleic acid), a pheromone carried by both males and females that increases courtship motivation and mating success (Dweck et al., 2015b; Lin et al., 2016; Zhuang et al., 2016). Furthermore, DmOr71a detects ethylphenols and governs the fly's preference for food enriched with healthy dietary antioxidants (Dweck et al., 2015a). In contrast to the labeled lines for pheromones, we call these “ecologically labeled lines,” meaning olfactory circuits tuned to specific ecologically relevant cues, whose activation leads to a predictable behavior, in this case feeding (Figure 1C; for an overview of examples of labeled lines and combinatorial coding see Table 1).

**Table 1:** Examples of behaviorally investigated coding systems in vinegar flies, hawkmoths, and mosquitoes.

**Species**	**Ors**	**Irs**	**Grs**	**Coding type**	**Receptor**	**Sensilla**	**Best ligand**	**Function**
*Drosophila melanogaster*	60	60	60^a^	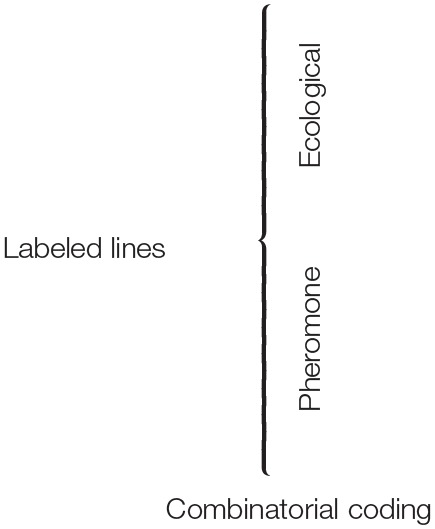	DmOr19a	ant. Ba.	limonene	Detecting oviposition sites^b^
					DmOr49a and Dm85f	ant. Ba.	iridomyrmecin	Enemy avoidance^c^
					DmOr56a	ant. Ba.	geosmin	Detecting toxic molds^d^
					DmOr71a	ant. Ba.	ethylphenols	Detecting antioxidants^e^
					DmOr47b	ant. Tr.	methyl laurate	Courtship motivation^f^
					DmOr67d	ant. Tr.	11-cis-vaccenyl acetatel	Aggregation, antiaphrodisiac^g^
					DmOr9a, 19a, 67a, 67b, 69a DmIr64a, 75a, *etc*.	ant. Ba.	acetoin,acetic acid, 2-phenyl ethanol	Balsamic vinegar^h^
*Manduca sexta*	73	21	45^i^	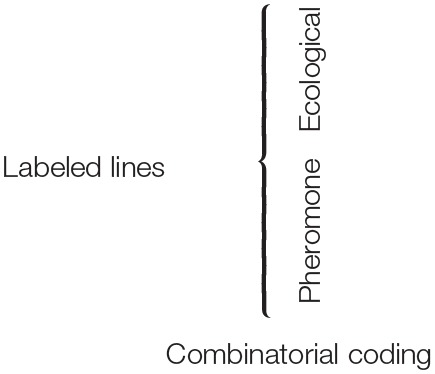	(MsGr1-2)	lab. Se	CO_2_	Evaluation of flower age^a^
					?	pro. St	benzyl acetone	Flower evaluation^k^
					MsOr1	ant. Tr.	bombykal	Male attraction^l^
					?	ant. Ba.	benzyl alcohol, benzaldehyde linalool	*Datura* flower^m^
*Anopheles gambiae*	73	46	60^n^	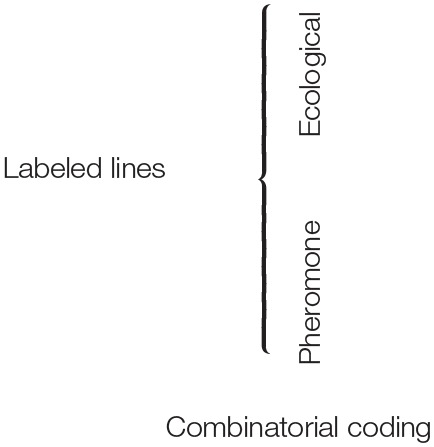	AgGr22-24	pal. Se.	CO_2_	Host detection^o^
					?	?	?	Mate recognition^p^
					?	?	ammonia, lactic acid, tetradecanoic acid	Host attraction^q^

Sensillum abbreviations: ant. Ba., antenna basiconica; ant. Tr., antenna trichodea; lab. Se, labial pit sensillum; pro.St, proboscis stylochonica; pal Se., maxillary palp capitate peg sensillum D. melanogaster:

a Joseph and Carlson, 2015;

b Dweck et al., 2013;

c Ebrahim et al., 2015;

d Dweck et al., 2015a;

e Stensmyr et al., 2012;

f Dweck et al., 2015b;

g Kurtovic et al., 2007;

h *Becher et al., 2010; Münch and Galizia, 2016*. *M. sexta:*

i Koenig et al., 2015;

j Guerenstein et al., 2004a,b;

k Haverkamp et al., 2016b;

l Wicher et al., 2017;

m *Riffell et al., 2009a,b, 2014*. *A. gambiae:*

n Kent et al., 2008; Liu et al., 2010; Pitts et al., 2011;

o Lu et al., 2007;

p unknown but hypothesized;

q *Smallegange et al., 2012*.

## The ecological function of labeled lines

When it comes to the detailed analysis of an ecologically labeled line, *Drosophila* might not be an ideal model species (Hansson et al., 2010), yet the ability to specifically silence OSNs of interest (or even activate them artificially in the absence of the corresponding ligand) still gives the fly a head start before potential other study systems. This targeted activation or deactivation of course helps to decipher, whether a specific neuronal circuit is necessary and sufficient to elicit a certain behavior and whether this circuit fulfills the criteria for an ecologically labeled line. However, such ecologically labeled lines are not restricted to food-related cues but are also involved in finding and/or judging suitable oviposition sites. OSNs expressing DmOr19a are tuned to limonene (an odor associated with citrus fruits) and their activation increases the female's probability to oviposit (Dweck et al., 2013). Contrary to that, OSNs expressing either DmOr56a, or coexpressing DmOr49a and DmOr85f detect geosmin or iridomyrmecin, respectively. While the former compound is a key odor of harmful bacteria and mold, the latter is the sex pheromone of a genus of parasitoid wasps (*Leptopilina*), which oviposit in—and thereby finally kill—*Drosophila* larvae. Hence, both odors are associated with danger for the offspring and, accordingly, the activation of the OSNs detecting these odorants results in a decreased oviposition motivation (Stensmyr et al., 2012; Ebrahim et al., 2015).

However, it is also easy to understand, why the fly does not only express highly specific receptors: if all its 60 Ors would be narrowly tuned, a fly—in the most extreme case—would detect only 60 compounds, i.e., only a negligible part of the olfactory world with its aliphatic acids, alcohols, aldehydes, esters, ketones, as well as chemicals with aromatic, alicyclic, polycyclic, and heterocyclic ring structures, and the substituted chemicals of each of these types. From our current knowledge, we can estimate that in *Drosophila* about a tenth of the Or repertoire is narrowly tuned, focusing on specific cues, while the rest of the Ors detects general features of the habitat (for a detailed description of the tuning properties of *Drosophila* Ors see Grabe et al., 2016). Why then does the fly invest such a significant part of its olfactory system into labeled lines? One hypothesis could be, that the processes needed for the combinatorial code are time consuming, and that signals of sex or danger need instant responses. However, flies respond to food odors in <200 ms (Bhandawat et al., 2010; Steck et al., 2012) although these odors are detected by broadly tuned receptors (Hallem and Carlson, 2006). It is, hence, rather unlikely that the detection of e.g., harmful mold and the resulting oviposition blockade, or the detection of cVA on mated females requires faster responses than that. Another hypothesis could be that combinatorial coding with broad receptors is less efficient than labeled lines in discriminating structurally similar odors. In this case the fly rather should trust in narrowly tuned receptors when signals definitely should not become misinterpreted like sex pheromones or parasitoid odors, but rather should depend on broadly tuned receptors to identify e.g., all kinds of rotten fruits. Moreover, it is conceivable that the additional computation by local interneurons, which might be necessary to establish a combinatorial odor code, introduces metabolic costs that an insect aims to avoid whenever possible to resort to a computationally simpler labeled line (Sterling and Laughlin, 2015).

The concept of labeled lines has originally been borrowed from the literature on taste processing in vertebrates and has been intensively studied in many different insect species over the last decades (Dethier, 1982). The direct link between such a line and a specific behavior makes the insect vulnerable to pest management for example through pheromone traps. Hence, the identification of labeled lines might help us to develop novel counterstrategies against insect disease vectors.

For hematophagous insects, carbon dioxide plays a crucial role in controlling host searching behavior, by gating the response to thermal targets and important human host odors (McMeniman et al., 2014). In the malaria mosquito, *Anopheles gambiae*, CO_2_ is detected by the gustatory receptors AgGr 22-24 (Lu et al., 2007). Interestingly, genetically modified mosquitoes that lack functional olfactory receptors, lose their attraction to general host odors but are still strongly attracted to CO_2_ (DeGennaro et al., 2013), showing that CO_2_ alone is sufficient to provoke host-finding behavior. This has led to the development of many models of CO_2_ baited mosquito traps already in the last century (Reeves, 1951). In addition, it will be interesting to test, whether mosquitoes also possess labeled lines for signals of danger like the mold-sensing and parasitoid-sensing ecologically labeled lines in *Drosophila* (Stensmyr et al., 2012; Dweck et al., 2013; Ebrahim et al., 2015), as this could facilitate the development of effective long-range repellents.

Moreover, labeled lines may not only be established through narrowly tuned receptors, but also by positioning olfactory receptors at the part of the insect body, which is closely linked to the task at hand. Recent studies in hawkmoths identified OSNs at the tip of the proboscis as well as on the ovipositor (Haverkamp et al., 2016b; Klinner et al., 2016). Due to their position these olfactory neurons are likely to convey exclusive information about a suitable feeding or oviposition place, even though these neurons have been shown to detect a variety of compounds relevant for their behavioral function. Similar olfactory neurons have also been found on the proboscis of the mosquitoes *A. gambiae* and *Aedes aegypti* (Jung et al., 2015; Riabinina et al., 2016), where neurons convey specific information about the quality of a host, making them promising targets for novel close-range repellents. Based on these studies, we suggest to broaden the definition of an ecologically labeled line by including neuronal circuits dedicated to a certain olfactory stimulus and very specific behavioral task, independent of the tuning breadth of their receptors.

## Odor detection in A chemically complex environment

In the present review, we do not enter into a discussion of the details on processing of positive or negative stimuli in higher brain centers as they were recently reviewed elsewhere (Sachse and Beshel, 2016), but rather discuss on how insects finally make use of the olfactory information. As already said, most of our knowledge regarding the neuronal and molecular basis of insect olfaction comes from *Drosophila*. Apart from this, scientists have today started to regard *Drosophila* not just as a model in a test tube but as an insect that evolved complex olfactory and behavioral strategies to cope with all kinds of environmentally-derived selection pressures. From our approaches we learned, that flies actively decide, whether or not to approach a food site or mating partner, or whether or not to drop eggs, and that they often do so based on olfactory cues. However, in spite of all the mentioned advantages of *Drosophila*, this fly feeds, mates, and oviposits within the rather narrowly defined world of a rotting fruit, which probably also limits the number of olfactory stimuli it has to process (Hansson et al., 2010). In contrast to this many other insects such as bees, mosquitoes, or moths may encounter a much wider and dynamic environment, which might then also require a higher degree of combinatorial coding. When it comes to olfactory-guided behavior, male Lepidoptera are probably the most famous example, as they locate ready-to-mate females over large distances just based on pheromone plumes. Apart from pheromone detection, olfaction is of course involved in many other tasks like host location (Proffit et al., 2015) and flower choice (Haverkamp et al., 2016a). Among the Lepidoptera, the hawkmoth *Manduca sexta* has developed into a major model species for insect neurophysiology, behavior, and ecology (Matsumoto and Hildebrand, 1981; Goyret et al., 2008; Riffell et al., 2013, 2014; Kessler et al., 2015; Sponberg et al., 2015; Levin et al., 2017).

Within its native range across North and South America *M. sexta* forages on a wide range of flowers from different plant species, which show the usual characteristics of the so the called “hawkmoth-pollination syndrome”: white corollas, slender corolla tubes and strong night-time volatile emissions (Riffell et al., 2013). Although very few, if any, compounds can be found that are present in all these flower bouquets, hawkmoth flowers still emit a characteristic blend of aromatic and terpenoid odors (Riffell et al., 2013). Hence, rather than focusing on a specific compound, the moths use a certain olfactory “Gestalt” to select a nectar resource or oviposition site (a phenomenon that was also observed in the malaria mosquito *A. gambiae* which targets odor combinations of hosts such as humans and cows, but not those of non-host species like chicken Majeed et al., 2016). In a series of experiments Riffell and his colleges reduced the blend complexity of a *Datura wrightii* flower, which normally contains 60–80 compounds to three essential volatiles: benzyl alcohol, benzaldehyde, and linalool (Riffell et al., 2009a,b, 2014). Notably though, the degree to which the moths were able to use this abstract representation of the *D. wrightii* flower bouquet was dependent on how chemically similar the odor background was to the target blend (Riffell et al., 2014; Figure 1A). These findings could indicate that the olfactory code, which an insect uses, might strongly depend on its olfactory environment i.e., in an environment that is olfactory very different from the odor of interest, few or even a single compound might be sufficient to provide reliable information, whereas a more complex representation might be required in an olfactory environment that is of lower contrast to the target odor. Interestingly, a slightly different form of background-dependent odor coding has been found in the malaria mosquito *Anopheles coluzzii* (*A. gambiae* sensu stricto molecular “M form”; van Loon et al., 2015). Here, it was shown that although under ambient CO_2_ conditions a three compound mix of ammonia, lactic acid, and tetradecanoic acid was sufficient to mimic a human host for blood-feeding (Smallegange et al., 2012), different other compounds could alter the attractiveness of this blend, but only under elevated CO_2_ levels (van Loon et al., 2015). Hence in case of the mosquito, CO_2_ probably helps the olfactory system to decide whether certain compounds belong to the general environment or to a potential host. Importantly, the ability of the mosquito to perceive a certain combination of odorants as a host blend might also be disrupted when artificial compounds are released from the same emission side as the natural blend. Verhulst and colleges for example found that isopropyl tetradecanoate, a compound commonly found in deodorants, significantly reduces the attraction of mosquitoes to the part of the human body to which the deodorant was applied (Verhulst et al., 2016), likely due to the formation of a novel odor blend, which was not recognized any longer by the mosquito as a host profile.

Two further experiments with hawkmoths taught us how complex such information gained from a combinatorial code can be: *M. sexta* exhibits an innate preference to the flower odors of *D. wrightii*, and (to a lower extend) to flowers of *Nicotiana attenuata*. This preference significantly increases, when one does not only present the floral odors alone, but presents them together with the leaf odors of the same plant. Hence, one could argue that both floral odors and leaf odors activate individual OSN populations and that the additive activation of whatever “positive” signals results in an increased attraction. However, the synergistic effect that was observed when odors from flowers and leaves of the same species were mixed, disappeared, when instead the flower and leaf odors from different species were mixed (Kárpáti et al., 2013). Similarly, ovipositing moths become highly attracted by leaf odors of *N. attenuata* and *D. wrightii*, but ignore the source as soon as both odors are mixed (Figure 1B; Spaethe et al., 2013). Obviously, it is not just a specific set of OSNs that, whenever it becomes activated, dictates the moth's behavior, but rather the combination of activated OSN populations that tell the moth, whether a scent is meaningful or not.

Combinatorial coding hence seems to be complex but highly important in the decision making of both moths and mosquitoes. Contrary to *Drosophila*, where several ecologically labeled lines have been identified (for a review see Mansourian and Stensmyr, 2015), the only so far fully unraveled labeled lines in these insects are involved in pheromone detection in case of the hawkmoth (Christensen and Hildebrand, 1987) or in CO_2_ detection in case of the mosquitoes. As with CRISPR/Cas9 (Doudna and Charpentier, 2014) finally molecular tools have emerged that e.g. can knockout genes of interest, future studies hopefully will reveal, whether the olfactory systems of *M. sexta*, mosquitoes and other insects follow the same logic like the *Drosophila* one, i.e., a functional combination of combinatorial code and ecologically labeled lines. However, to fully tackle these questions, it will also be necessary to follow the animal into its natural environment and to analyze the nervous system while it is dealing with real life challenges. Although, such undertakings might still appear far off, the advancements made in recording and stimulating neurons of freely flying dragonflies and hawkmoth (Hinterwirth et al., 2012; Thomas et al., 2012), have also shown us that analyzing the insect brain in its natural habitat might in fact already be in reach (Dickinson, 2015).

But how could such knowledge be applied? The understanding of labeled lines, their ligands, and their behavioral impact has already helped us to better control agricultural pest insects. Sex pheromones for example are widely used for trapping and mating disruption both in crop production and food storage (Reisenman et al., 2016), while at the same time protecting the important ecosystem function of many insects, such as pollination (Klein et al., 2007). Apart from crop-related issues, the world is facing huge problems due to insect vectors, such as mosquitoes, spreading diseases like Malaria or Zika. So far no long-distance sex pheromones in mosquitoes have been identified that could be used for trapping and/or mating disruption. However, any identified ecologically labeled line and its corresponding ligand could open up new possibilities for controlling insect pests and disease vectors as well as for supporting insect ecosystem services such as pollination or pest control.

## Author contributions

All authors contributed to the outline of this review. AH and MK: wrote the first draft of the manuscript and all authors contributed to the revision.

### Conflict of interest statement

The authors declare that the research was conducted in the absence of any commercial or financial relationships that could be construed as a potential conflict of interest.
